# FBXO45 restricts HIV-1 replication by inducing SQSTM1/p62-mediated autophagic degradation of Tat

**DOI:** 10.1128/jvi.01912-24

**Published:** 2025-02-12

**Authors:** Mingxiu Xu, Haobo Hu, Weijing Yang, Jiaxiang Zhang, Hong Wang, Wenyan Zhang, Chen Huan

**Affiliations:** 1 Department of Infectious Diseases, Infectious Diseases and Pathogen Biology Center, The First Hospital of Jilin University 117971 , Changchun, Jilin, China; 2 Institute of Virology and AIDS Research, The First Hospital of Jilin University 664674 , Changchun, Jilin, China; 3 Key Laboratory of Organ Regeneration and Transplantation of The Ministry of Education, The First Hospital of Jilin University 117971 , Changchun, Jilin, China; University Hospital Tübingen, Tübingen, Germany

**Keywords:** Tat, FBXO45, HIV-1, transcription, autophagic degradation

## Abstract

As a key regulator of human immunodeficiency virus type 1 (HIV-1) transcription, Tat plays an essential role in viral replication and latency, making it a promising target for designing viral control strategies. Identifying host factors that modulate Tat and exploring the underlying mechanisms will benefit our understanding of HIV-1 transcriptional regulation and provide valuable insights into Tat-based therapeutic strategies. Here, by employing the TurboID approach, we discovered high-affinity binding between FBXO45 and Tat. Our findings demonstrate that FBXO45 negatively regulates Tat by promoting Tat ubiquitination and directing it to autophagic degradation. Autophagic degradation of Tat has been reported, but the specific underlying mechanisms remain unidentified. We elucidated this issue by providing evidence that FBXO45-mediated Tat polyubiquitination is an essential prerequisite for this process. Silencing of FBXO45 leads to a deficiency of autophagy receptor SQSTM1/p62 to bind and facilitate the autophagic degradation of Tat. Our results further underscore the crosstalk between post-translational modifications of Tat by demonstrating that the phosphorylation site of the Tat S62 residue is required for ubiquitination induced by FBXO45. Furthermore, in the context of the regulation of HIV-1, FBXO45 inhibits viral replication and maintains the latency of HIV-1 by suppressing viral transcription. Importantly, FBXO45 overexpression significantly attenuated viral rebound after antiretroviral therapy withdrawal. In summary, our findings suggest a novel role for FBXO45 in regulating HIV-1 replication by inducing the ubiquitination and SQSTM1/p62-dependent autophagic degradation of Tat. Considering the indispensable role of Tat in the regulation of HIV-1 replication and reactivation, FBXO45 may be a potential target for therapeutic intervention against HIV-1.

**IMPORTANCE:**

HIV-1 Tat plays an indispensable role in regulating viral transcription and is a promising target for achieving a functional cure for AIDS. Identifying the host factors that modulate Tat expression could benefit the development of anti-HIV-1 strategies targeting Tat. Using TurboID assay, we identified a significant interaction between FBXO45 and Tat. Functionally, FBXO45 ubiquitinates and directs Tat for SQSTM1/p62-mediated autophagic degradation, thereby effectively restricting HIV-1 replication and maintaining HIV-1 latency by suppressing Tat-dependent viral transcription. These findings uncover a novel role for FBXO45 in regulating Tat and broaden our understanding of the host mechanisms involved in Tat processing.

## INTRODUCTION

HIV-1 transcription is a pivotal step in the replication cycle, and Tat plays an indispensable role in facilitating efficient transcription and maintaining elevated levels of viral replication (1, 2). Following the infection of CD4+ T cells or macrophages, HIV-1 RNA undergoes reverse transcription and integrates into the host chromatin as a provirus, which is subsequently transcribed by host RNA polymerase II (RNAP II) (3). Despite the successful initiation of transcription by the RNAP II complex, the resulting short viral transcripts are unable to complete efficient viral replication. To overcome this limitation, HIV-1 encodes the transcriptional transactivator Tat, which hijacks the positive transcriptional elongation factor b (p-TEFb) complex composed of cyclin T1 (CCNT1) and cyclin-dependent kinase 9 (CDK9) into the transactivation response element (TAR) of the HIV-1 long terminal repeat (LTR) promoter (3–6). This leads to the phosphorylation of the DRB sensitivity-inducing factor (DSIF), negative elongation factor (NELF), and the C-terminal domain (CTD) of RNAP II. Consequently, stalled polymerase is released, resulting in significant enhancement of HIV-1 transcriptional elongation (7, 8). Therefore, Tat establishes a robust positive feedback loop that amplifies a cascade of transcriptional activation signals and exponentially increases the efficiency of HIV-1 transcription (9, 10).

Because of the crucial role of Tat, an increasing number of studies have been conducted to elucidate the regulatory mechanisms that modulate Tat. Notably, ubiquitination, a prevalent post-translational modification involved in virus-host interactions, has been implicated in the processing of Tat (11–13). For instance, Tat is recruited by the host lncRNA NRON to interact with CUL4B and PSMD11 and facilitates polyubiquitination and subsequent degradation through the proteasomal pathway (14). In contrast, the host lncRNA uc002yug.2 downregulated CUL4B and PSMD11, thereby inhibiting Tat degradation (15). Host UHRF1 promotes K48-linked ubiquitination-mediated proteasomal degradation of Tat while also competing with p-TEFb for binding to HIV-1 Tat, leading to the inhibition of viral transcription and establishment of viral latency (16). Tat also undergoes selective autophagy-mediated degradation (17). These observations suggest that Tat regulation is a multifaceted process involving various host factors, indicating the significant potential for further investigation.

To gain a comprehensive interpretation of Tat regulation, we employed TurboID, a highly efficient and sensitive method combined with mass spectrometry, to identify potential host factors involved in modulating Tat. Using this approach, we successfully identified the high-affinity binding of FBXO45 to Tat and found that FBXO45 negatively regulates Tat. Notably, the autosome-lysosome inhibitor Bafilomycin A1 (BafA1), rather than the proteasome inhibitor MG132, effectively blocked the FBXO45-mediated degradation of Tat, indicating that FBXO45 degradation of Tat is dependent on the autosome-lysosome pathway. Previous studies have demonstrated the involvement of autophagic degradation in Tat processing (17). However, the specific factors contributing to this process, apart from the autophagic receptor SQSTM1/p62, remain unidentified. In the present study, we further elucidated this by demonstrating that FBXO45 is required for Tat autophagic degradation. Silencing of FBXO45 significantly attenuated the interaction between SQSTM1/p62 and Tat, implying that FBXO45-induced Tat ubiquitination is a prerequisite for SQSTM1/p62 binding and triggering autophagic degradation. Furthermore, FBXO45 overexpression remarkably suppressed HIV-1 replication and viral rebound after antiretroviral therapy (ART) withdrawal, highlighting the significance of FBXO45 as a potential target for counteracting HIV-1. Taken together, these findings demonstrate a novel regulatory role for FBXO45 in Tat modulation, which enhances our understanding of Tat regulation and benefits the development of Tat-based therapeutic strategies against HIV-1.

## RESULTS

### TurboID and mass spectrometry identified FBXO45 as a potential regulator of HIV-1 Tat

**Fig 1:**
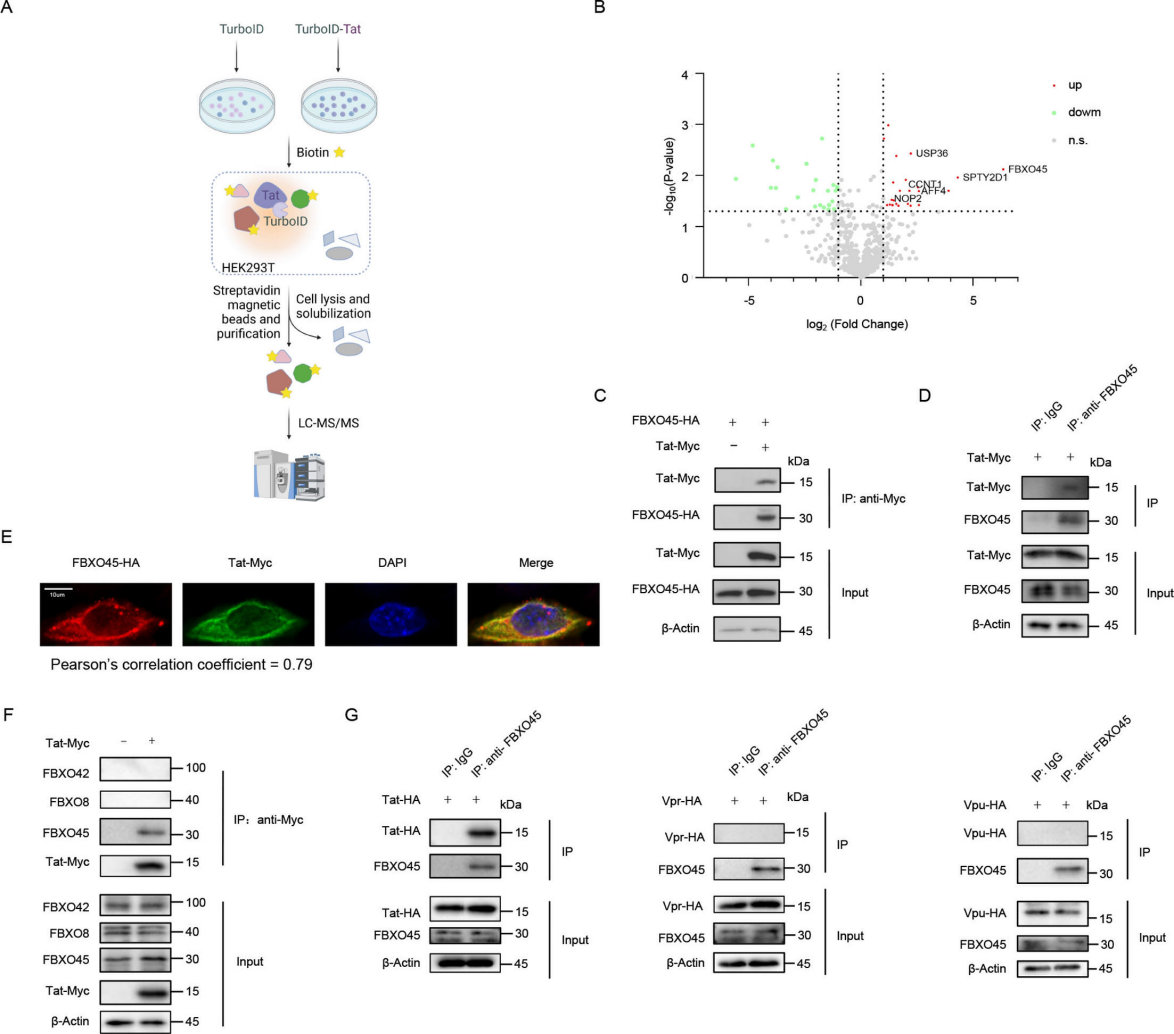
TurboID and mass spectrometry identified FBXO45 as a potential regulator of Tat. (**A)** Schematic diagram of TurboID-based identification of Tat-proximal proteins. (**B)** Volcano plot presenting the differentially biotinylated proteins in HEK293T cells transfected with the TurboID-Tat over TurboID empty control vector. The dashed lines indicate a *P* value of 0.05 and a fold change of 2 on *Y* and *X* axis, respectively. (**C)** Co-IP confirmed the interaction between Tat and FBXO45. FBXO45-HA was transfected with control or Tat-Myc into HEK293T cells, and then, the cells were subjected to Myc IP and immunoblotting. (**D)** Endogenous FBXO45 interacted with Tat-Myc. HEK293T cells transfected with Tat-Myc were subjected to co-IP with IgG or anti-FBXO45 antibody, followed by immunoblotting analysis with indicated antibodies. (**E)** FBXO45 co-localized with Tat. HeLa cells seeded onto poly-L-lysinecoated microscope slides were transfected with the indicated constructions. Anti-HA antibody was used to immunostain FBXO45-HA (red), Tat-Myc was immunostained with anti-Myc in green, and the nucleus was stained with DAPI (blue). Cells were observed under a Zeiss LZM710 confocal microscope, Bars, 10 µm, Pearson’s correlation coefficient was analyzed using the ImageJ software. (**F-G)** The interaction between Tat and FBXO45 was specific. (**F)** HEK293T cells transfected with control or Tat-Myc were subjected to Myc IP, followed by immunoblotting analysis with antibodies against FBXO45, FBXO8, or FBXO42. (**G)** HIV-1 protein Tat-HA, Vpr-HA, or Vpu-HA were co-transfected into HEK293T cells. Cell lysates were collected 48 hours post-transfection and subjected to co-IP using IgG or anti-FBXO45 antibody, followed by immunoblotting analysis. The schematic workflow in (A) is generated using BioRender (http://biorender.com/).

Similar to other viral proteins of HIV-1, Tat is tightly regulated by host factors through complex mechanisms (11). Given the essential role of Tat in HIV-1 replication, elucidating the host factors involved in the regulation of Tat could facilitate the development of anti-HIV-1 therapies targeting Tat. We used a biotin proximity labeling system (18) to identify the host factors that regulate Tat. The promiscuous biotin ligase TurboID was fused to Tat on its N terminal for the precise and temporal biotinylation of surrounding proteins, which could be enriched by streptavidin (Fig. 1A).

By performing mass spectrometry, we identified putative Tat interactors (fold change >2; *P* < 0.05) that exhibited enrichment in TurboID-Tat over the TurboID empty control vector. Among them, well-known Tat interactors, such as CCNT1 (6) and AFF4 (19), were identified, reflecting the efficiency and specificity of the TurboID assay utilized in the present study. Notably, FBXO45 exhibited the most significant enrichment among the Tat-proximal proteins (Fig. 1B), indicating that FBXO45 may interact with and regulate Tat. To confirm this, we validated the interaction between FBXO45 and Tat in HEK293T cells. Co-immunoprecipitation (Co-IP) assays revealed that both ectopically overexpressed (Fig. 1C) and endogenous FBXO45 (Fig. 1D) co-immunoprecipitated with Tat. In line with these observations, confocal microscopy further showed that Tat colocalized with FBXO45 in HeLa cells with the Pearson’s correlation coefficient of 0.79 (Fig. 1E). Further investigations were conducted to confirm the specificity of the Tat-FBXO45 interaction. As shown in Fig. 1F and G, the binding of Tat to FBXO45 was not observed with other F-box proteins, such as FBXO8 or FBXO42 (Fig. 1F). When FBXO45 was used as the bait, other HIV proteins, such as Vpr and Vpu, failed to be co-immunoprecipitated (Fig. 1G), indicating that the Tat-FBXO45 interaction is highly specific. Collectively, these findings provide novel evidence for the interaction between Tat and FBXO45, which is highly suggestive of the role of FBXO45 in the regulating of Tat.

### FBXO45 negatively regulates HIV-1 Tat stability through autosome-lysosome degradation

**Fig 2:**
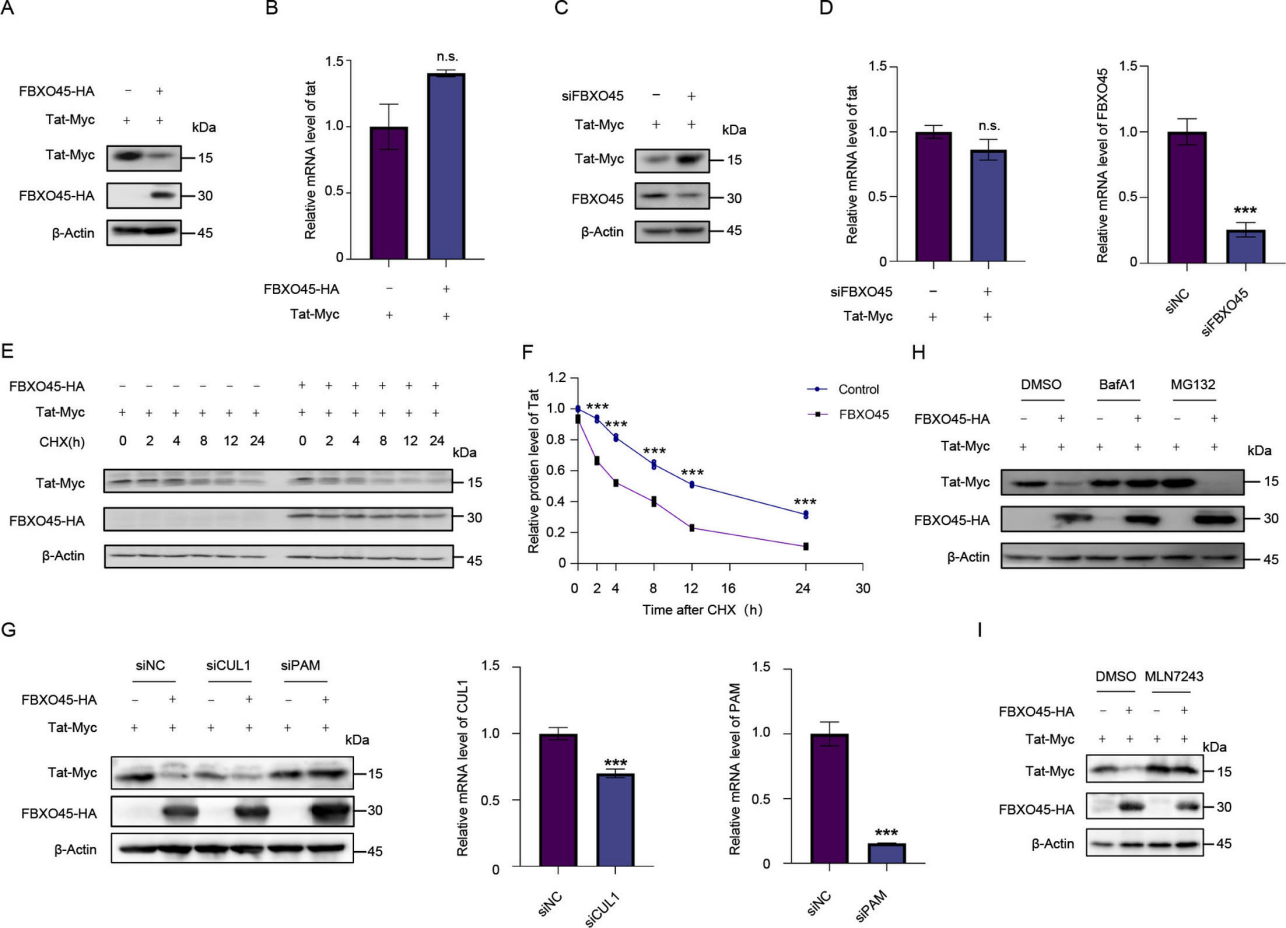
FBXO45 negatively regulates Tat stability through autosome-lysosome degradation. (**A-B)** Ectopic expression of FBXO45 decreased Tat levels without affecting its mRNA abundance. HEK293T cells were transfected with Tat-Myc plus control or FBXO45-HA plasmids. Forty-eight hours later, the cells were harvested, and the levels of indicated proteins were detected by immunoblotting (**A**). The mRNA levels of *tat* were quantified with RT-qPCR assays (**B**).**(C-D)** Silencing of FBXO45 increased Tat levels without affecting its mRNA abundance. HEK293T cells were transfected with Tat-Myc plus scramble siRNA or siRNA targeting FBXO45. Forty-eight hours later, the cells were harvested, and the levels of indicated proteins were detected by immunoblotting (**C**). The mRNA levels of *tat* and FBXO45 were quantified with RT-qPCR assays (**D**).**(E-F)** FBXO45 impaired the stability of Tat. HEK293T cells were cotransfected with Tat-Myc and FBXO45-HA or control vectors. Twenty-four hours post-transfection, the cells were treated with protein synthesis inhibitor CHX (100 µg/mL) or dimethyl sulfoxide (DMSO) as control, and then harvested at indicated time points for immunoblotting analysis. (**G)** Silencing of PAM-impaired FBXO45-induced Tat degradation. HEK293T cells were transfected with Tat-Myc plus siRNA targeting PAM or CUL1 in the absence or presence of FBXO45-HA. The levels of indicated proteins were analyzed by immunoblotting, and mRNA levels of CUL1 and PAM were detected by RT-qPCR. (**H)** FBXO45-mediated Tat degradation through autosome-lysosome pathway. Tat-Myc and FBXO45 plasmids were co-transfected into HEK293T cells for 32 h and then treated with either the proteasomal inhibitor MG132 (10 µM), the autophagy-lysosome inhibitor BafA1 (10 nM), or DMSO as control for additional 16 h. Then, the cells were collected for immunoblotting with indicated antibodies.** (I)** FBXO45-mediated Tat degradation is ubiquitin-dependent. Tat-Myc and FBXO45 plasmids were co-transfected into HEK293T cells for 32 h and then treated with the ubiquitin-activating enzyme inhibitor MLN7243 (0.5 uM) or DMSO as control for additional 16 h. Then, the cells were collected for immunoblotting with indicated antibodies. *P* values were calculated using the two-tailed student’s *t*-test, ***P < 0.001, and n.s. denotes no significance.

FBXO45 functionally serves as an adaptor to facilitate the assembly of the SCF E3 complex, thereby promoting polyubiquitination and degradation of substrates. Therefore, we investigated whether FBXO45 regulates Tat levels. To this end, we cotransfected HEK293T cells with Tat plus control or FBXO45 expression vectors and observed a significant decrease in Tat levels upon overexpression of FBXO45 (Fig. 2A). However, the *tat* mRNA levels remained unaffected (Fig. 2B). Conversely, when endogenous FBXO45 was silenced using siRNA, the level of Tat increased remarkably, but leaving its mRNA unchanged (Fig. 2C and D). These data strongly indicate that FBXO45 exerts a negative effect on Tat through post-translational modifications. To validate these findings, we performed the cycloheximide (CHX) chase assay. As shown in Fig. 2E and F, compared with the control group, FBXO45 overexpression shortened the half-life of Tat when protein synthesis was inhibited by CHX, further substantiating that FBXO45 promotes Tat degradation.

It is worth noting that FBXO45 is an exception to other F-box protein family members, as it does not form an E3 complex with typical CUL1 due to its E42R substitution (20). Instead, the Ring-finger protein PAM is hijacked by FBXO45 to degrade substrates (20–22). Thus, we investigated whether PAM was required for FBXO45 to regulate Tat. As shown in Fig. 2G, when endogenous PAM, rather than CUL1, was knocked down, FBXO45 lost its ability to degrade Tat, implying that PAM is involved in Tat regulation mediated by FBXO45.

Tat has been reported to undergo degradation via the proteasomal (14, 16, 23, 24) or autosome-lysosome pathway (17). To investigate the specific mechanism involved in the FBXO45-mediated degradation of Tat, cells transfected with Tat and FBXO45 were treated with either MG132 or BafA1, which inhibits the proteasome or autosome-lysosome pathway, respectively. DMSO was used as a negative control. Notably, the results demonstrated that treatment with BafA1 effectively reversed the decrease in Tat induced by FBXO45 (Fig. 2H), indicating that FBXO45 mediates Tat degradation via the autosome-lysosome pathway. We further treated the cells transfected with Tat and FBXO45 with an inhibitor of the ubiquitin-activating enzyme (UAE), MLN7243. As shown in Fig. 2I, treatment with 0.5 µM MLN7243 blocked FBXO45-mediated Tat degradation, implying that FBXO45 regulates Tat via a ubiquitin-dependent mechanism.

### FBXO45 ubiquitinates HIV-1 Tat and facilitates its SQSTM1/p62-dependent autophagic degradation

**Fig 3:**
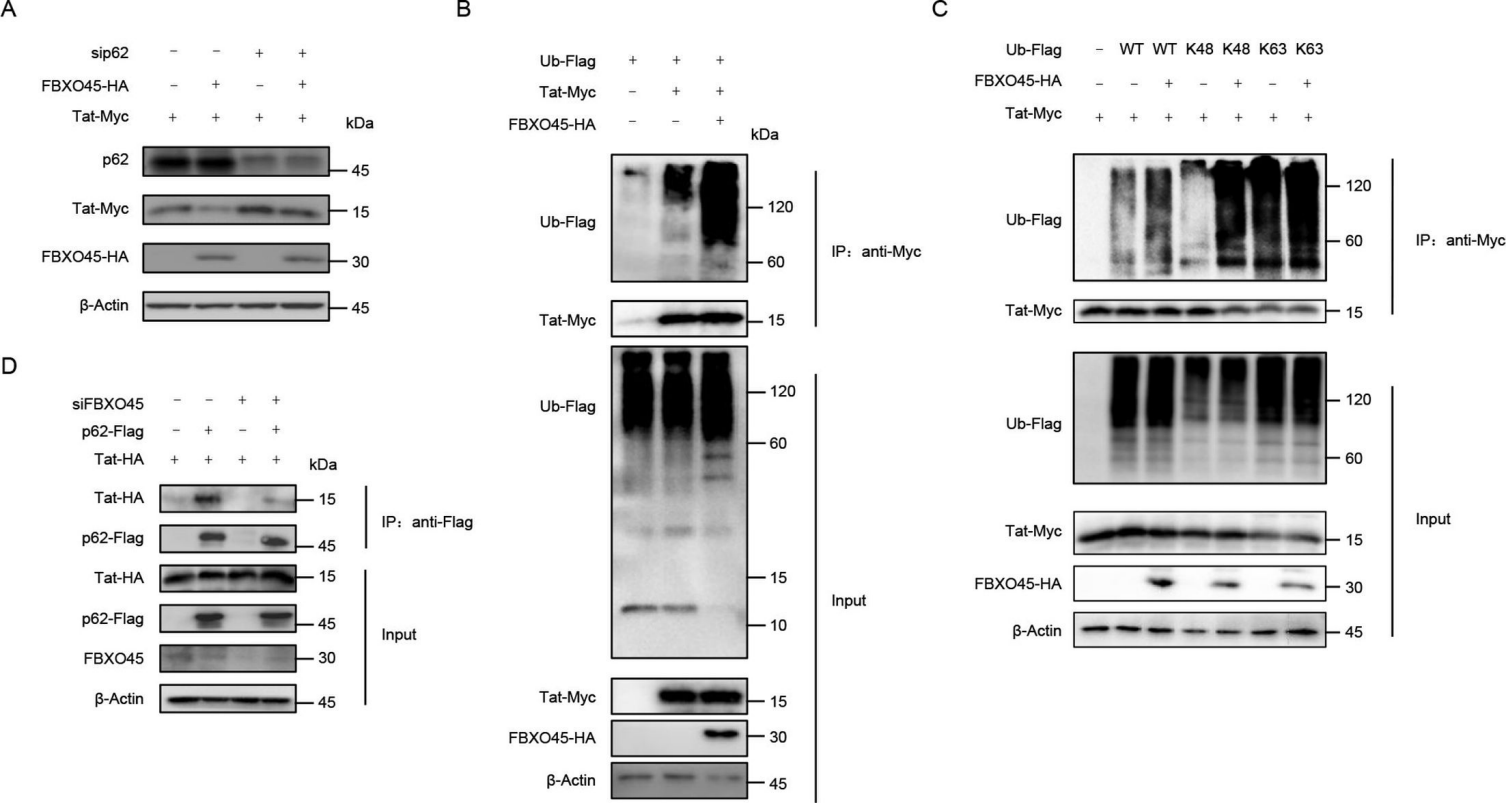
FBXO45 ubiquitinates Tat and facilitates its SQSTM1/p62-dependent autophagic degradation. (**A)** Silencing p62 restored the FBXO45-induced degradation of Tat. HEK293T cells were transfected with Tat-Myc plus siRNA targeting p62 or non-targeting control siRNA in the absence or presence of FBXO45-HA. The levels of indicated proteins were analyzed by immunoblotting. (**B)** FBXO45 facilitates Tat ubiquitination. HEK293T cells were transfected with the indicated constructs and treated with 10 nM BafA1 for 16 h before harvesting. At 48 h after transfection, cell lysates were subjected to co-IP with anti-Myc antibodies conjugated to agarose beads followed by immunoblotting with the indicated antibodies. (**C)** FBXO45 promotes K48-linked polyubiquitination of Tat. HEK293T cells transfected with indicated constructs were treated with 10 nM BafA1 for 16 h before harvesting. At 48 h post-transfection, cell lysates were subjected to Myc IP, followed by immunoblotting analysis. (**D)** Knockdown of FBXO45 impaired the interaction between Tat and p62. HEK293T cells were transfected with Tat-HA plus siRNA targeting FBXO45 or non-targeting control siRNA in the absence or presence of p62-Flag and treated with 10 nM BafA1 for 16 h before harvesting. At 48 h after transfection, cell lysates were subjected to co-IP with anti-Flag antibodies conjugated to agarose beads followed by immunoblotting with the indicated antibodies.

The autophagy receptor SQSTM1/p62 has been reported to contribute to the autophagic degradation of Tat (17), which prompted us to investigate whether p62 was required for FBXO45-induced Tat degradation. As shown in Fig. 3A, when endogenous p62 was silenced with siRNA, the degradation of Tat induced by FBXO45 was restored (Fig. 3A, lanes 3–4 vs lanes 1–2), suggesting that FBXO45 degrades Tat in a p62-dependent manner, further confirming the involvement of autophagy in the FBXO45-induced degradation of Tat.

Autophagic degradation typically requires substrate ubiquitination (25), and our observations in Fig. 2I demonstrate the involvement of ubiquitin in the FBXO45-mediated regulation of Tat. Therefore, we examined whether FBXO45 promotes Tat ubiquitination to facilitate p62-mediated autophagic degradation. As shown in Fig. 3B, FBXO45 overexpression significantly promoted Tat ubiquitination. Furthermore, considering the specific chain type of ubiquitin, FBXO45 facilitated the K48-linked polyubiquitination of Tat (Fig. 3C). These *in vivo* ubiquitination results imply that by ubiquitinating Tat, FBXO45 may provide a prerequisite for p62 to facilitate the autophagic degradation of Tat. To confirm this, we examined the effect of FBXO45 on the interaction between p62 and Tat using co-IP assays. Using Flag-tagged p62 as bait, we observed that p62 immunoprecipitated with Tat (Fig. 3D, lane 2). However, when FBXO45 was knocked down using siRNA, the interaction between p62 and Tat was disrupted (Fig. 3D, lane 4), further verifying the indispensable role of FBXO45 involved in p62-mediated autophagic degradation of Tat. Based on these findings, we conclude that the FBXO45-induced polyubiquitination of Tat is essential for p62 to facilitate the autophagic degradation of Tat.

### Functional domains of FBXO45 for ubiquitinating HIV-1 Tat

**Fig 4:**
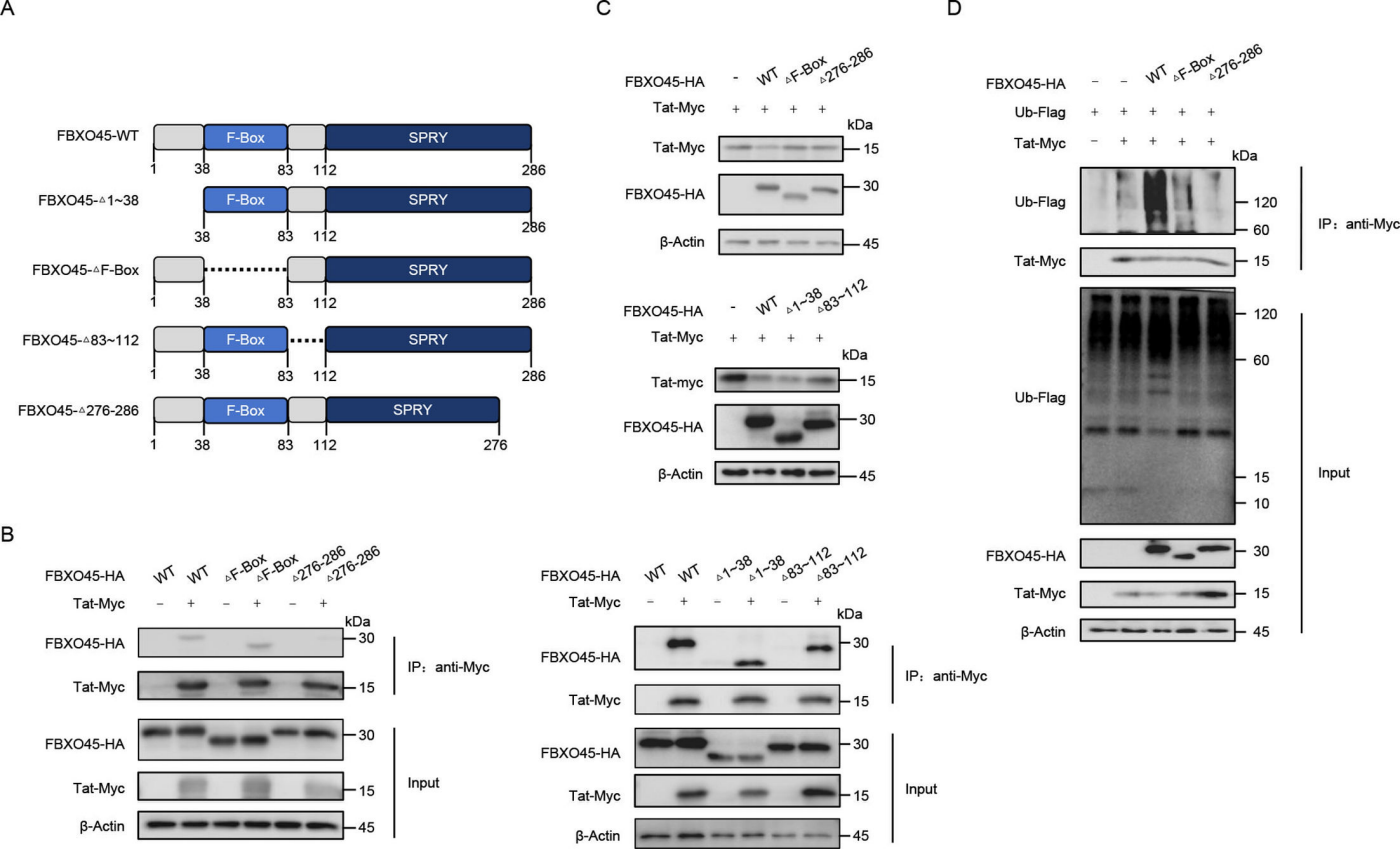
Functional domains of FBXO45 for ubiquitinating Tat. (**A)** Schematic of FBXO45 domains and its mutant construct. (**B)** Deletion mutations in the SPRY domain disrupt FBXO45 binding to Tat. HEK293T cells were transfected with indicated plasmids and treated with 10 nM BafA1 for 16 h before harvesting. At 48 h after transfection, the cell lysates were subjected to co-IP with anti-Myc antibodies conjugated to agarose beads, followed by immunoblotting with the indicated antibodies. (**C)** FBXO45 lacking F-Box or containing impaired SPYR domain lost the ability to degrade Tat. HEK293T cells were transfected with Tat-Myc plus wildtype or mutant plasmids of FBXO45-HA. Forty-eight hours later, the cells were harvested, and the levels of indicated proteins were detected by immunoblotting. (**D)** Intact F-Box and SPYR domains are required for FBXO45 to ubiquitinate HIV-1 Tat. HEK293T cells were transfected with indicated plasmids. At 32 h post-transfection, the cells were treated with 10 nM BafA1 for additional 16 h and subjected to Co-IP with anti-Myc antibodies conjugated to agarose beads followed by immunoblotting with the indicated antibodies.

Having observed the regulatory role of FBXO45 in Tat degradation, we mapped the functional domains of FBXO45 to elucidate the underlying mechanism. The F-Box domain (residues 38–83) and SPRY domain (residues 112–286) of FBXO45 have been proven to be responsible for its interaction with SCF E3 complex and substrates (26, 27). Therefore, we evaluated their contributions, as well as those of the remaining domains, in regulating Tat by generating deletion mutants to delete residues 1–38 (Δ1–38), the F-Box (ΔF-Box), and residues 83–112 (Δ83–112) or disrupt the folded structure of the SPRY domain (Δ276–286), as reported previously (28) (Fig. 4A). Notably, when the SPRY domain was disrupted, FBXO45 lost its ability to bind Tat (Fig. 4B), further confirming its substrate-binding ability. Furthermore, FBXO45 lacking the F-Box domain or containing an impaired SPRY domain resulted in a deficiency in Tat degradation (Fig. 4C), indicating that both the F-Box and SPRY domains are indispensable for FBXO45 to regulate Tat. In addition, we examined whether these functional domains were essential for FBXO45 to ubiquitinate Tat. By employing an *in vivo* ubiquitination assay, we found that when lacking the F-Box domain or containing an impaired SPRY domain, FBXO45 lost its Tat ubiquitination-promoting ability (Fig. 4D lanes 4 and 5), which was observed in wild-type FBXO45 (Fig. 4D lane 3). Collectively, these results suggest that intact F-Box and SPRY domains are required for FBXO45 to regulate HIV-1 Tat.

### Phosphorylation site S62 of HIV-1 Tat is required for ubiquitination and degradation by FBXO45

**Fig 5:**
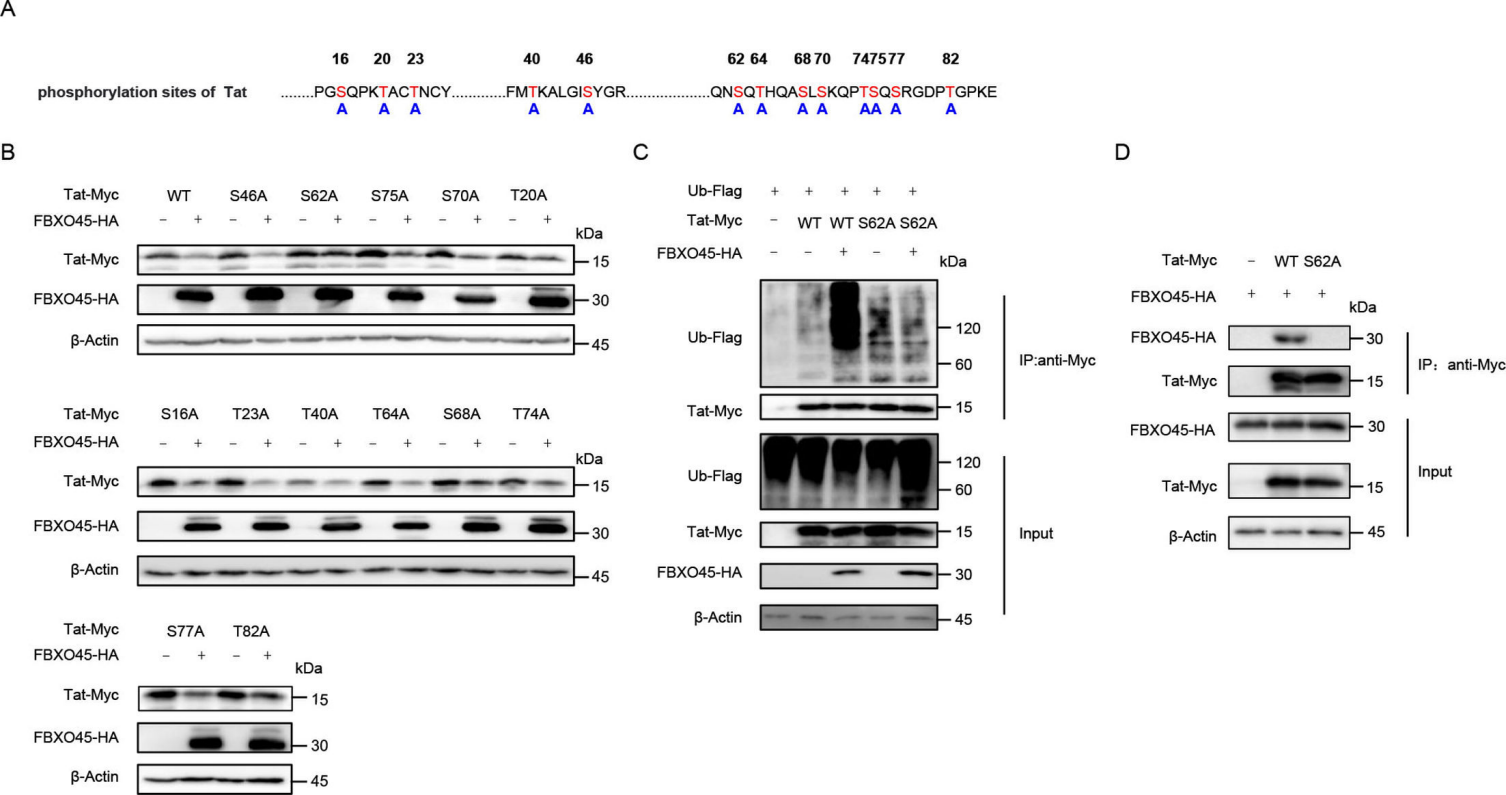
Phosphorylation site S62 of Tat is required for FBXO45 to ubiquitinate and degrade Tat (**A)** Schematic of phosphorylation sites in Tat. (**B)** S62A substitution in Tat was resistant to the degradation induced by FBXO45. HEK293T cells were transfected with wild-type or phosphorylation site mutations of Tat-Myc plus control or FBXO45-HA plasmids. Forty-eight hours later, the cells were harvested, and levels of indicated proteins were detected by immunoblotting. (**C)** S62A substitution in Tat impaired the ubiquitination-promoting activity of FBXO45 and was treated with 10 nM BafA1 for 16 h before harvesting. HEK293T cells were transfected with indicated plasmids for 48 h and subjected to co-IP with anti-Myc antibodies conjugated to agarose beads, followed by immunoblotting with the indicated antibodies. (**D)** Residue S62 of Tat is required for Tat-FBXO45 interaction. HEK293T cells were transfected with indicated plasmids for 48 h and subjected to Myc IP followed by immunoblotting analysis.

Substrate phosphorylation is required by some, but not all, F-box family members to catalyze substrate ubiquitination (29, 30). Therefore, we evaluated whether this scenario could also be employed by FBXO45 to facilitate Tat degradation. Previously identified phosphorylation sites (31–33) and other serine or threonine residues within Tat were substituted with alanine to abrogate the potential phosphorylation of the corresponding sites (Fig. 5A). These constructs were subsequently co-transfected with either the control or FBXO45 expression vector. As demonstrated in Fig. 5B, compared with the wild type, the Tat S62A substitution was resistant to degradation induced by FBXO45, implying that phosphorylation of S62 was required for FBXO45 to exert its Tat degradation function. To further validate this observation, we conducted an *in vivo* ubiquitination assay and found that the Tat ubiquitination-promoting activity of FBXO45 was abolished by substitution of residue S62 of Tat with A (Fig. 5C, lanes 5 vs 3). In contrast to the wild-type, the S62A mutant of Tat failed to co-immunoprecipitate FBXO45 (Fig. 5D). Taken together, these results demonstrate that phosphorylation site S62 of Tat is required for FBXO45 to ubiquitinate and degrade Tat.

### FBXO45 inhibits HIV-1 replication by suppressing viral transcription

**Fig 6:**
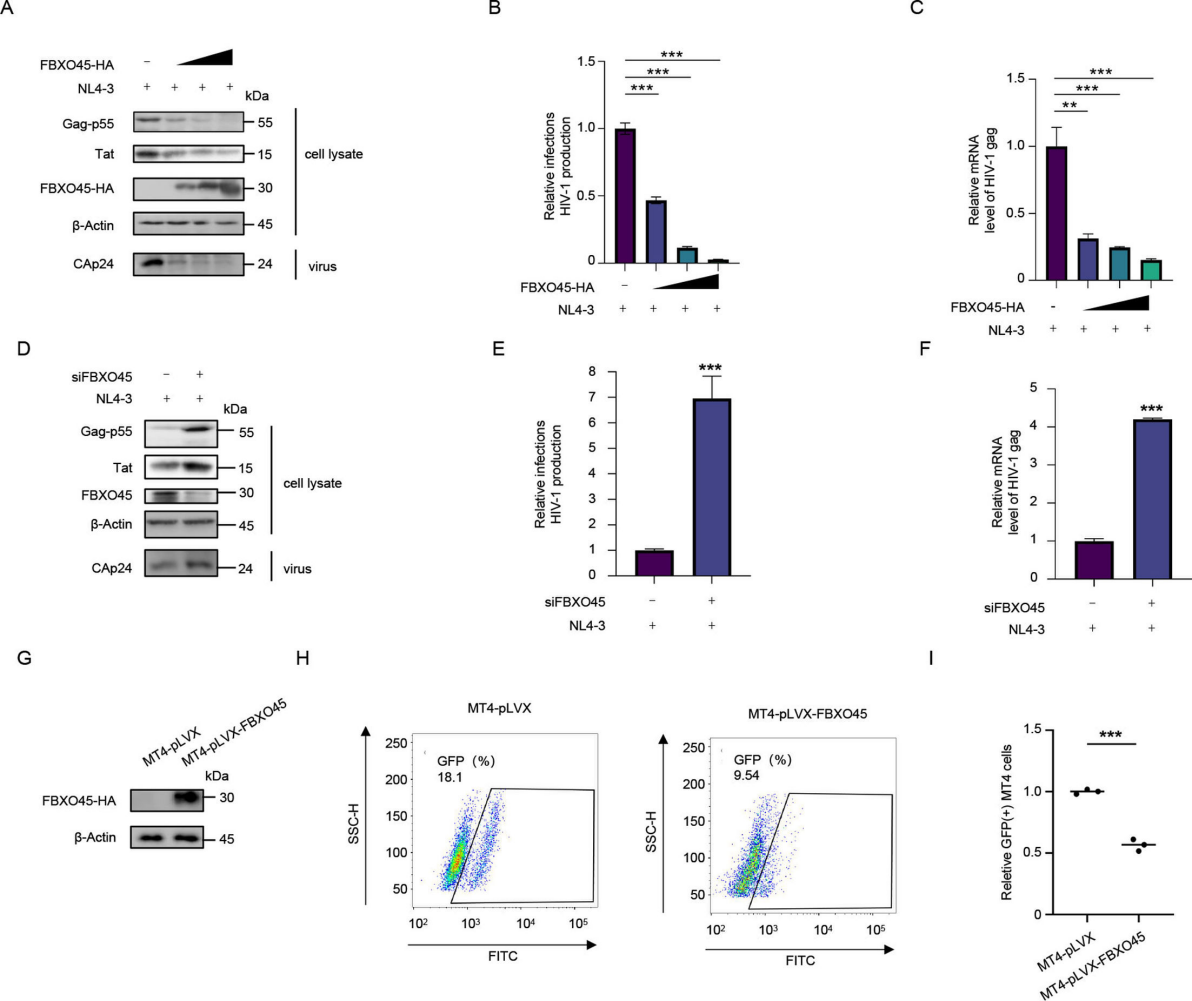
FBXO45 inhibits HIV-1 replication. (**A-C)** FBXO45 inhibits HIV-1 replication in HEK293T cells. The HEK293T cells were co-transfected with pNL4-3 viral vector plus an increased dose of FBXO45-HA, and the cells and culture supernatant were harvested for immunoblotting with the indicated antibodies at 48 h post-transfection (**A**). Then, the TZM-bl cells were infected with the culture supernatant for additional 48 h, and the yield of HIV-1 was quantified by measuring the luciferase activity. The yield of virus derived from the cells transfected with pNL4-3 plus control vector was set as 1 (**B**). The mRNA level of HIV-1 *gag* was analyzed by RT-qPCR, and the *gag* mRNA level of cells transfected with pNL4-3 plus control vector was set as 1 (**C**). **(D-F)** Silencing of FBXO45 enhances HIV-1 replication in HEK293T cells. The HEK293T cells were co-transfected with pNL4-3 viral vector plus siRNA against FBXO45, and the cells and culture supernatant were harvested for immunoblotting with the indicated antibodies at 48 h post-transfection (**D**). Then, the TZM-bl cells were infected with the culture supernatant for additional 48 h, and the yield of HIV-1 was quantified by measuring the luciferase activity. The yield of virus derived from the cells transfected with pNL4-3 plus control siRNA was set as 1 (**E**). The mRNA level of HIV-1 *gag* was analyzed by RT-qPCR, and the *gag* mRNA level of cells transfected with pNL4-3 plus control siRNA was set as 1 (**F**). **(G-I)** FBXO45 inhibits HIV-1 replication in MT-4 cells. MT-4 cells stably over-expressing FBXO45 and their parental cells were constructed (**G**) and infected with HIV-1 NL4-3-EGFP virus. The GFP-positive cells were measured by flow cytometry at 48 h post-infection (**H**). The ratio of GFP-positive cells in stably over-expressing FBXO45 MT-4 cells relative to that in control MT-4 cells was calculated (**I**). *P* values were calculated using the two-tailed student’s *t*-test (**E, F, and I**) or ANOVA (**B and C**). **P < 0.01, ***P < 0.001.

Given the crucial role of Tat in HIV-1 transcription and virion production, the observation that FBXO45 facilitated Tat degradation prompted us to investigate whether FBXO45 regulates HIV-1 replication. To explore this, we transfected HEK293T cells with NL4-3 and increased the amount of FBXO45. FBXO45 significantly inhibited HIV-1 replication in a dose-dependent manner, as evidenced by intracellular Gag-p55 and Tat levels, and CAp24 in supernatant (Fig. 6A), infectious virion yield (Fig. 6B), and intracellular HIV-1 *gag* mRNA levels (Fig. 6C). In contrast, when endogenous FBXO45 was silenced using siRNA, HIV-1 replication was markedly enhanced (Fig. 6D through F). Moreover, we confirmed the anti-HIV activity of FBXO45 in T cells. MT-4 cells stably overexpressing FBXO45-HA were constructed (Fig. 6G) and infected with NL4-3-GFP virus. Consistently, the ectopic expression of FBXO45 significantly reduced the susceptibility of MT-4 cells to HIV-1, as determined by measuring the percentage of GFP-positive cells (Fig. 6H and I). In summary, these findings demonstrate a significant inhibitory effect of FBXO45 on HIV-1 replication.

**Fig 7:**
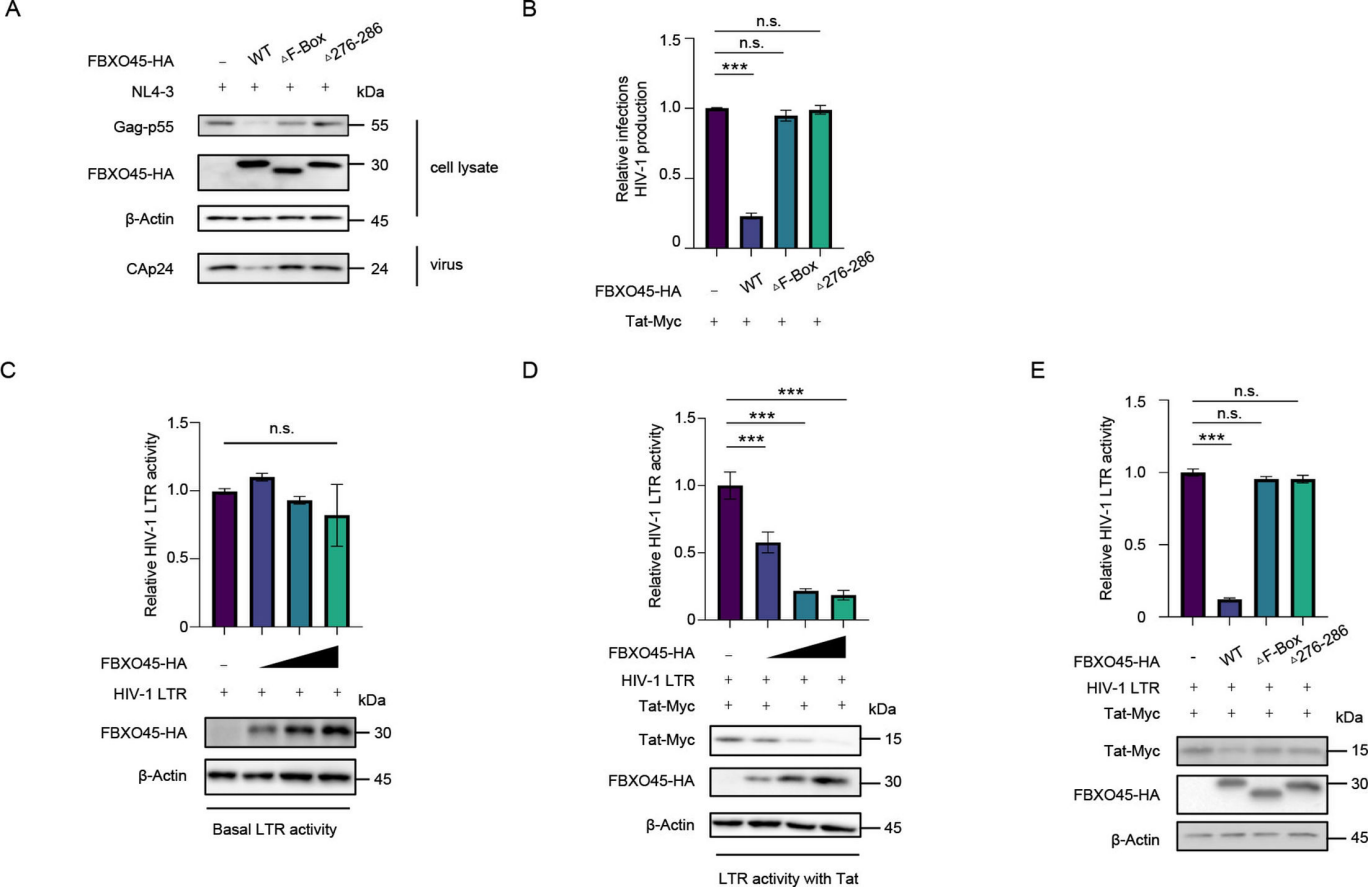
FBXO45 suppresses Tat-dependent transcription of HIV-1. (**A-B)** FBXO45 lacking F-Box or SPRY domains exhibited inability to inhibit HIV-1. The HEK293T cells were co-transfected with pNL4-3 viral vector plus wild-type or mutants of FBXO45-HA, and the cells and culture supernatant were harvested for immunoblotting with the indicated antibodies at 48 h post-transfection (**A**). Then, the TZM-bl cells were infected with the culture supernatant for additional 48 h, and the yield of HIV-1 was quantified by measuring the luciferase activity. The yield of virus derived from the cells transfected with pNL4-3 plus control vector was set as 1 (**B**). **(C-D) **FBXO45 suppresses HIV-1 LTR activity in a Tat-dependent manner. HEK293T cells were transfected with HIV-1 LTR-luciferase construct plus increasing doses of FBXO45 in the absence (**C**) or presence of Tat (**D**) for 48 h, and LTR activity and protein levels were measured. (**E)** FBXO45 lacking F-Box or containing an impaired SPRY domain could not suppress HIV-1 LTR activity. The HEK293T cells were co-transfected with HIV-1 LTR vector plus wild-type or mutants of FBXO45-HA. The cells were harvested for immunoblotting and LTR activity detection at 48 h post-transfection, with the activity of cells transfected with HIV-1 LTR plus control vector set as 1. *P* values were calculated using ANOVA, ***P < 0.001 and n.s. denotes no significance.

In addition, to further investigate the functional domains of FBXO45, we transfected HEK293T cells with NL4-3 and wild-type or truncated constructs of FBXO45; the empty vector VR1012 served as a control. As shown in Fig. 7A and B, compared with wild-type, FBXO45 lacking the F-Box domain or containing an impaired SPRY domain resulted in an impaired capacity to inhibit HIV-1, which is consistent with their inability to degrade Tat.

Considering the essential role of Tat in HIV-1 transcription, we examined the potential of FBXO45 in modulating viral transcription. HEK293T cells were transfected with the HIV-1 LTR luciferase construct and increasing doses of FBXO45 in the absence (Fig. 7C) or presence of Tat (Fig. 7D). Notably, FBXO45 significantly attenuated Tat-induced enhancement of HIV-1 LTR activity in a dose-dependent manner (Fig. 7D), whereas basal LTR activity in the absence of Tat remained unaffected (Fig. 7C), indicating that FBXO45 exerts its anti-HIV function by suppressing Tat-mediated amplification of viral transcription. To further validate these findings, we introduced the truncated mutations of FBXO45, which lost their ability to degrade Tat and suppress HIV-1. Intriguingly, the lack of the F-Box domain or the presence of an impaired SPRY domain abolished the suppressive effect of FBXO45 on LTR activity (Fig. 7E). Collectively, these findings corroborate that FBXO45 exerts its anti-HIV effects by suppressing viral transcription through Tat degradation.

### High level of FBXO45 reinforces HIV-1 latency and prevents viral rebound upon ART withdrawal

**Fig 8:**
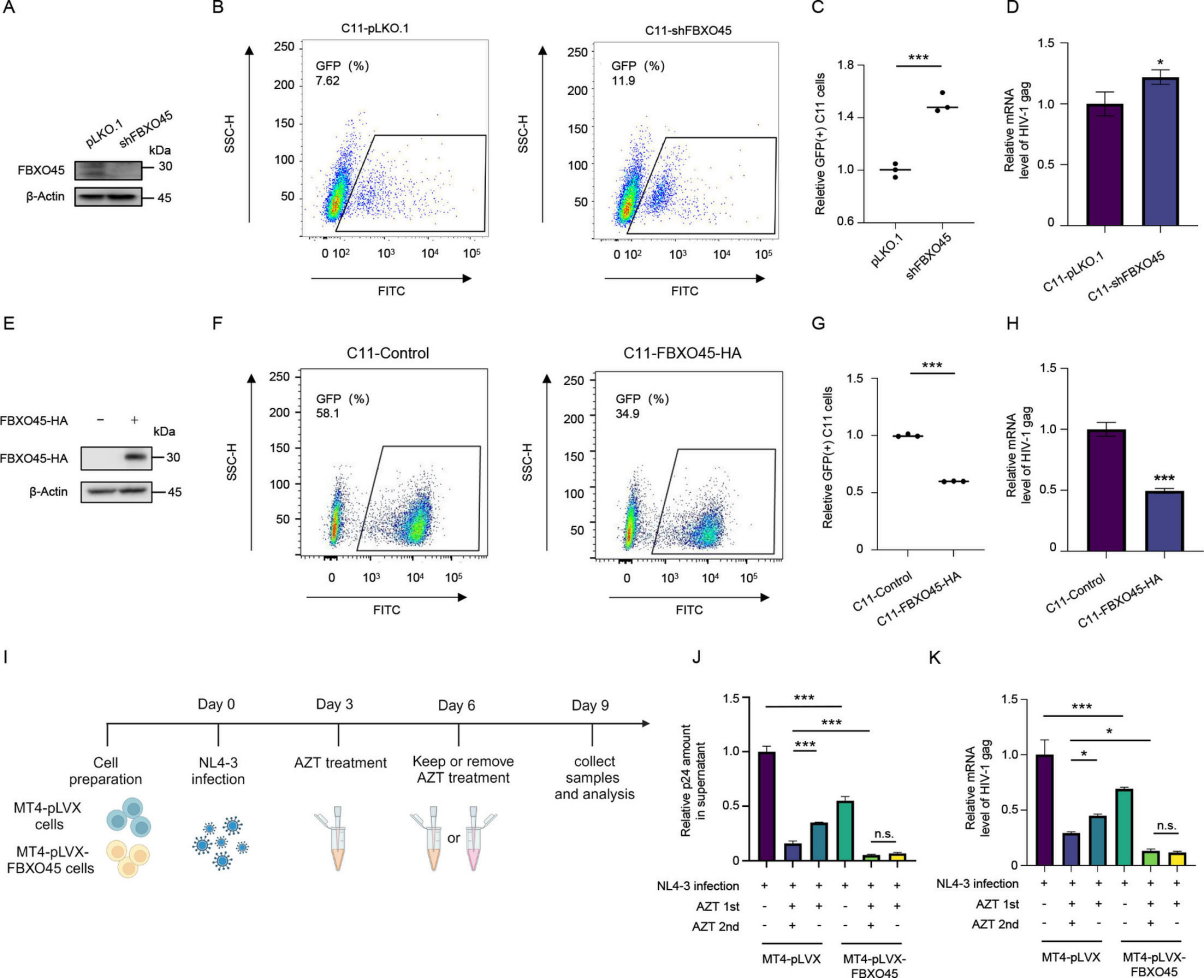
High level of FBXO45 reinforces HIV-1 latency (**A-D).** Silencing of FBXO45 activates latent HIV-1 replication in C11 cell lines. The endogenous FBXO45 in C11 cells was knocked down by using FBXO45-specific shRNA (**A**). Then, the GFP-positive cells of FBXO45-sh or control C11 cells were detected by flow cytometry (**B**). The relative ratio of GFP-positive cells as in panel B was calculated (**C**). The mRNA level of HIV-1 *gag* was analyzed by RT-qPCR (**D**). **(E-H**) Overexpression of FBXO45 attenuates latent HIV-1 reactivation in C11 cell lines. FBXO45-overexpressing C11 cells were established (**E**), and the GFP-positive cells (**F-G**) and mRNA level of HIV-1 *gag* were detected (**H**). **(I-K**) High level of FBXO45 suppresses latent HIV-1 rebound. Experimental design for the analysis of HIV-1 replication in AZT-treated MT-4 cells expressing FBXO45 or control cells (**I**). Control and stably overexpressing FBXO45 MT-4 cells were infected with HIV-1 for 3 days. Subsequently, the cells were treated with AZT (20 µM) for an additional 3 days, followed by being switched into either AZT-free or AZT-containing medium for another period of 3 days. On day 9, the cells were harvested, the amount of p24 antigen in the supernatant was quantified by ELISA assay (**J**), and mRNA level of HIV-1 *gag* was analyzed by RT-qPCR (**K**). The schematic workflow in (**I)** is generated using BioRender (http://biorender.com/). *P* values were calculated using the two-tailed student’s *t*-test (**C, D, G, and H**), and ANOVA (**J and K**). *P < 0.05, ***P < 0.001, and n.s. denotes no significance.

Suppression of viral transcription promotes HIV-1 latency (34). Thus, the inhibitory effect of FBXO45 on HIV-1 LTR-driven gene expression suggests that it may play a role in maintaining HIV-1 latency. To further investigate this issue, we used C11, a Jurkat-derived cell line latently infected with HIV-1, and detected the association between FBXO45 levels and maintenance or reactivation of HIV-1 latency. A lentivirus containing an shRNA targeting FBXO45 was used to infect C11 cells, and stable silencing of endogenous FBXO45 in C11 cells was achieved through subsequent screening (Fig. 8A). As shown in Fig. 8B through D, the reduction of endogenous FBXO45 resulted in the significant reactivation of latent HIV-1, by comparing the GFP (+) cells (Fig. 8B and C), as well as viral transcription (Fig. 8D) of control and FBXO45 stably silencing C11 cell lines. In contrast, when FBXO45 was overexpressed in C11 cells, the reactivation of the latent virus induced by phorbol 12-myristate 13-acetate (PMA) treatment was significantly attenuated (Fig. 8E through H). Collectively, these results support the hypothesis that elevated FBXO45 levels reinforce HIV-1 latency by inhibiting viral transcription.

In addition, we detected the regulatory effect of FBXO45 in the context of viral rebound, a clinically significant consequence following the discontinuation of anti-HIV therapy. As shown in Fig. 8I, we employed a strategy to simulate the clinical manifestations of drug withdrawal leading to HIV-1 rebound (35, 36). Briefly, control and stably overexpressing FBXO45 MT-4 cells were infected with HIV-1 for 3 days. Subsequently, the cells were treated with azidothymidine (AZT) for an additional 3 days before switching to either AZT-free or AZT-containing medium for another 3 days. By quantifying the viral load in the supernatant and HIV-1 *gag* mRNA levels in cells using ELISA and RT-qPCR, respectively, we observed significant inhibition of HIV-1 replication upon FBXO45 overexpression (Fig. 8J and K, lane 4 vs lane 1). Furthermore, this anti-HIV effect was potentiated by AZT treatment (Fig. 8J and K, lanes 2 and 5). Importantly, interruption of AZT treatment resulted in a less pronounced viral rebound in MT-4 cells expressing FBXO45 than in control cells (Fig. 8J and K, lanes 5–6 vs lanes 2–3). Taken together, these results suggest that FBXO45 increases HIV-1 latency and attenuates viral rebound upon discontinuation of ART.

## DISCUSSION

By forming a powerful positive transcriptional feedback loop through specific interactions with host factors and TAR, HIV-1 Tat functions as a key regulatory factor for viral transcription. Downregulating the level of Tat or breaking the Tat-mediated transcriptional enhancement almost equals turning off the “switch” of viral transcription, which will effectively reinforce the virus into silence and achieve the goal of a functional cure. Based on this concept, “block and lock” strategy has been postulated, and several attempts to lock HIV-1 into persistent latency by counteracting Tat have been established. For instance, didehydro-corticostatin A (dCA), which specifically binds to the unstructured basic region of Tat to disrupt Tat-TAR interactions and restrict RNAP II recruitment to the HIV-1 promoter, has been demonstrated to delay and reduce viral rebound upon treatment interruption (37–39). In addition, the small molecule Q308 has been reported to silence the HIV-1 provirus and reduce the size of the viral reservoir by inhibiting Tat-mediated viral transcription (40). Thus, these observations illustrate that strategies targeting Tat may be beneficial for achieving a functional cure for AIDS.

**Fig 9:**
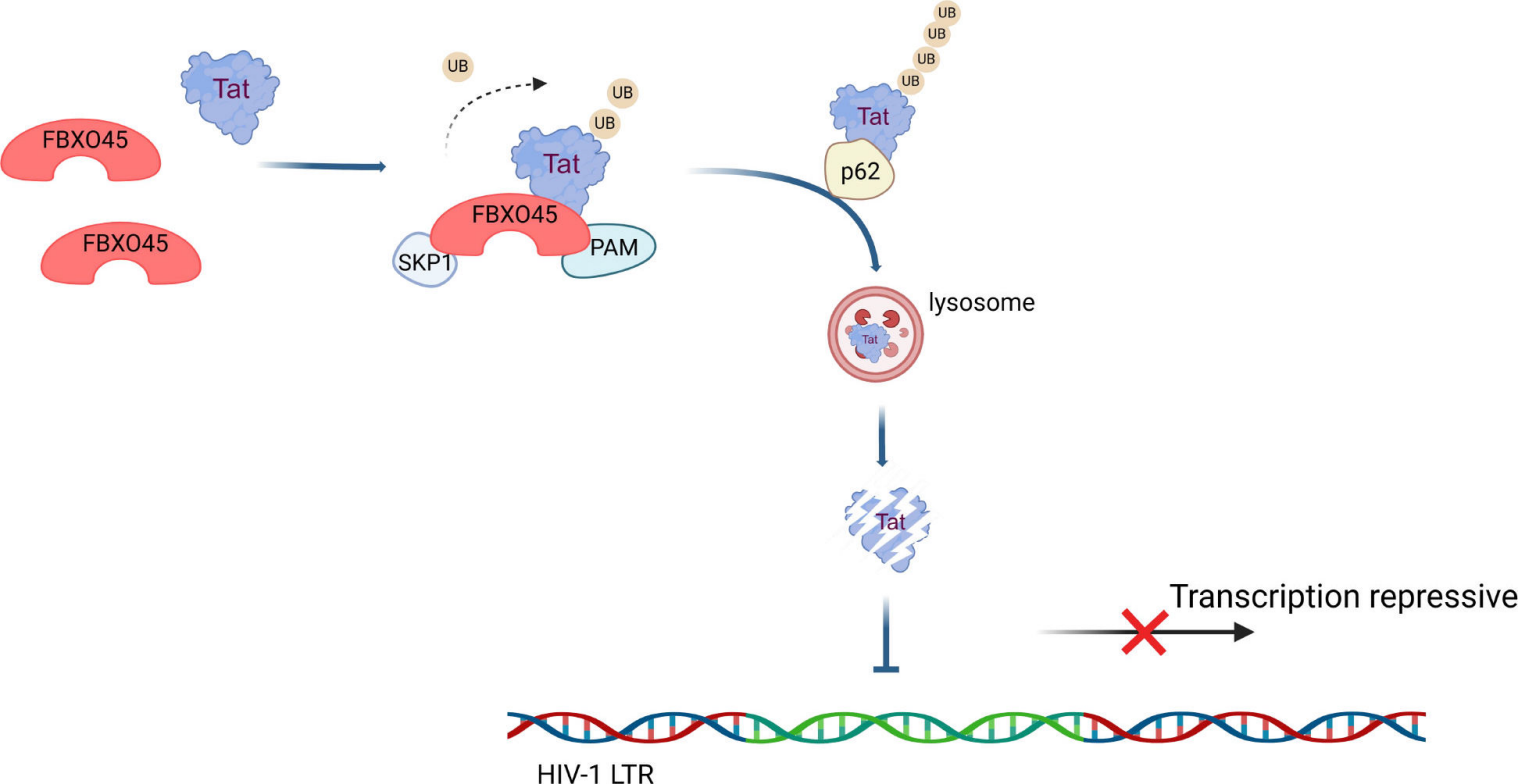
Schematic of the molecular mechanisms underlying the function of FBXO45 in regulating HIV-1. As a substrate-recognition subunit of SCF E3 ubiquitin ligase complex, FBXO45 interacts with HIV-1 Tat via its SPRY domain. FBXO45 promotes polyubiquitination of Tat and triggers p62-dependent autophagic degradation, thereby inhibiting HIV-1 replication by suppressing viral transcription. The schematic model is generated using BioRender (http://biorender.com/).

With this concept in mind, we used the TurboID assay to identify potential regulators of Tat and found that FBXO45 ubiquitinated and directed Tat to SQSTM1/p62-mediated autophagic degradation, thereby restricting HIV-1 replication and maintaining HIV-1 latency by suppressing Tat-dependent viral transcription (Fig. 9). Serving as a substrate-recognition subunit of SCF E3 ligase, FBXO45 and other F-box family members have been demonstrated to be involved in biological and pathological processes, ranging from cancinogenesis (41–43) to nervous system (20–22) and psychiatric disorders (44), and their potential as therapeutic targets has been underscored (45). With an increasing number of proteins identified as substrates regulated by F-box proteins, a significant contribution of F-box proteins to viral infection has been recognized. FBXO45 was recently shown to be involved in influenza infection by promoting IFNLR1 ubiquitination and degradation (46), suggesting that FBXO45 may exert potential regulatory functions in virus-host interactions. More recently, another F-box protein, FBXO34, was reported to activate latent HIV-1 by ubiquitinating and degrading HNRNP-U, leading to the release of viral translation (47). In the present study, we identified FBXO45 as a new regulator of HIV-1 Tat and provided evidence that FBXO45 exerts latency, indicating the intricate involvement of F-box proteins in regulating HIV-1 latency at multiple levels.

Substrate phosphorylation has been proven to be necessary for many SCF E3 ligases to ubiquitinate and degrade substrates (29, 30). For FBXO45, Wu et al. recently reported that the phosphorylation of USP39 enhances the ubiquitination and degradation induced by FBXO45 (26). We then investigated whether this is applicable to FBXO45-mediated Tat regulation and found that Tat ubiquitination triggered by FBXO45 requires Tat S62 phosphorylation. The Tat S62A mutant has been reported to exhibit weakened phosphorylation (33), and the ubiquitination and degradation-promoting effects induced by FBXO45 were attenuated upon this substitution (Fig. 5). The crosstalk between multiple post-translational modifications is a prevalent regulatory mechanism that controls vital cellular processes (48–50). Residue S62 of Tat is reported to be phosphorylated by PKR, leading to an increase in the interaction between Tat and TAR and enhancement of viral transcription (51). In the present study, phosphorylated S62 facilitated the ubiquitination and degradation of Tat, suggesting that there may be regulatory machinery that maintains a balance between Tat protein abundance and its transcriptional activity.

The ubiquitin system plays an indispensable role in the host-virus interplay (52–54). For instance, HIV-1 Vif hijacked CUL5 to form the SCF E3 ligase machinery, which degrades and counteracts the restriction of A3G (55). In addition to the ability of viral proteins to utilize the ubiquitin system to counteract host restrictions, viral proteins can also be subjected to this regulatory system. HIV-1 Tat, a small protein containing multiple lysine residues, has been proven to undergo ubiquitination modification. Apart from its established role in regulating Tat transcriptional activation (56–58), ubiquitination also directs Tat to be recognized and degraded by the proteasome (12, 14–16). In contrast to the extensively investigated ubiquitin-proteasome pathway, research on the involvement of autophagy in Tat processing is limited. In the present study, we demonstrated that Tat undergoes autophagic degradation, as evidenced by the autophagy inhibitor BafA1 treatment blocked FBXO45-mediated degradation of Tat. Consistent with a previous finding that the autophagy receptor p62 mediates Tat autophagic degradation (17), we found that p62 silencing attenuated FBXO45-directed Tat degradation. Notably, we further elucidated Tat ubiquitination induced by FBXO45 is required for p62 to bind to and trigger Tat for autophagic degradation. These observations provide comprehensive evidence regarding the autophagic degradation of Tat.

In addition to FBXO45, several other candidate factors interacting with Tat were identified using the TurboID assay in the present study. Among them, the presence of well-established Tat interactors such as CCNT1 (6), AFF4 (19), and NOP2 (59), which was recently reported to suppress HIV-1 transcription by competing with Tat for TAR binding, validates the efficacy of the TurboID assay employed here. In addition, the deubiquitinase USP36 exhibited a significant ability to bind Tat. Considering that Tat has been recently shown to undergo deubiquitination and that deubiquitination can both stabilize and downregulate Tat (13, 60), whether and how USP36 is involved in this process should be further elucidated.

As an essential process in the regulation of the viral life cycle, viral transcription has been emphasized for its profound role in manipulating latency. The present study uncovered a novel role for FBXO45 in suppressing HIV-1 transcription by degrading Tat. Our findings also showed that FBXO45 maintained HIV-1 latency and attenuated the viral rebound after ART withdrawal. Considering the essential role of Tat in HIV-1 replication and latency, an analog or activator of FBXO45 may be a promising HIV-1 latency-promoting agent (LPA) and has advantages for the functional cure of AIDS. Collectively, these findings shed light on host-virus interactions and expand our understanding of latency establishment.

## MATERIALS AND METHODS

### Identification of host factors interacting with Tat

The HEK293T cell lines were transfected with the TurboID-Tat or TurboID and cultured for 18–24 h. Subsequently, a final concentration of 500 µM biotin was added to the cell culture medium. After a 10-min labeling, the cells were halted by placing them on ice and underwent five washes with ice-cold PBS. Gentle pipetting of PBS onto the cells facilitated detachment from petri dishes, followed by collection of pellets through centrifugation at 1,500 × *g* for 3 min. The supernatant was discarded, and the resulting pellet was lysed in 1.2 mL RIPA lysis buffer (50 mM Tris pH 8, 150 mM NaCl, 0.1% SDS, 0.5% sodium deoxycholate, 1% Triton X-100, 1× protease inhibitor cocktail, and 1 mM PMSF) by gentle pipetting and incubating at 4°C for 5 min. Lysates were clarified twice through centrifugation at 10,000 × *g*, each time lasting for a duration of 10 min at a temperature of 4°C.

To enrich biotinylated samples for proteomic analysis, 50 µL of streptavidin-coated beads (slurry) were subjected to two washes with RIPA buffer and subsequently incubated overnight at 4°C with clarified cell lysates under rotation. The beads were subsequently washed twice with 1 mL of RIPA lysis buffer, once with 1 mL of 1 M KCl, once with 1 mL of 0.1 M Na2CO3, once with 1 mL of 2 M urea in 10 mM Tris-HCl (pH 8.0), and twice with 1 mL RIPA lysis buffer. Biotinylated proteins were then eluted from the beads by boiling them in a solution containing 60 µL of protein loading buffer (2× concentration) supplemented with 20 mM DTT and 2 mM biotin. Then the samples were analyzed by mass spectrum.

### Plasmid construction

FBXO45 cDNA was cloned into the VR1012 expression vector with HA Tag for transient transfection or into the pLVX-IRES-neo (Clontech Laboratories Inc., San Francisco, CA, USA) lentiviral vector to construct the stably overexpressing FBXO45 cells. Mutagenesis assay was employed to construct FBXO45 plasmids bearing F-Box deletion (ΔF-Box), residues 1–38 deletion (Δ1–38), residues 83–112 deletion (Δ83–112), or impaired SPRY domain (Δ276–286). The plasmids pNL4-3, pNL4-3-ΔEnv-GFP were obtained from the AIDS Research and Reference Reagents Program, Division of AIDS, National Institute of Allergy and Infectious Diseases (NIAID), National Institutes of Health (NIH). Tat-Myc, p62-Flag, and HIV-1-LTR-luciferase were constructed as previously described (15, 61).

The primers used for plasmid construction are documented in Table 1, and all constructs employed in the present study were confirmed by sequencing.

**TABLE 1:** Primers used for plasmid construction

Primer name	Primer direction	Sequence (5’−3’)
FBXO45-HA	Forward	ATGTACCCATACGATGTTCCA
FBXO45-HA	Reverse	TCAGCCATCCAGGGGCTTGCC
FBXO45-△276–286	Forward	TACCGAGGTGACCTGAGGATCCAGATC
FBXO45-△276–286	Reverse	AGGTCACCTCGGTATTGCCGTAC
FBXO45-△F-Box	Forward	GCTCTGGAAGCGGAGCCTGCGCCAGATCCC
FBXO45-△F-Box	Reverse	AGGCACCGCTTCCAGAGCCGGCGCCGGC
Tat-S16A	Forward	GAAGCACCCTGGG GCT CAGCCAAAGACAGC
Tat-S16A	Reverse	CCCCAGGGTGCTTCCAAGGCTCCAG
Tat-T20A	Forward	GTCCCAGCCAAAGGCTGCTTGCACAAACTG
Tat-T20A	Reverse	CCTTTGGCTGGGACCCAGGGTGCT
Tat-T23A	Forward	CAAAGACAGCTTGCGCTAACTGCTACTGCAAG
Tat-T23A	Reverse	CGCAAGCTGTCTTTGGCTGGGACCC
Tat-T40A	Forward	AGGTGTGCTTCATGGCTAAGGCTCTGGGCAT
Tat-T40A	Reverse	CCATGAAGCACACCTGGCAGTGGAAGC
Tat-S46A	Forward	GGCTCTGGGCATTGCTTACGGACGCAAGAAG
Tat-S46A	Reverse	CAATGCCCAGAGCCTTTGTCATG
Tat-S62A	Forward	CGCTCACCAGAACGCTCAGACACACCAAGC
Tat-S62A	Reverse	CGTTCTGGTGAGCGCGTCTCCTCT
Tat-T64A	Forward	ACCAGAACTCTCAGGCTCACCAAGCGTCCC
Tat-T64A	Reverse	CCTGAGAGTTCTGGTGAGCGCGTC
Tat-S68A	Forward	GACACACCAAGCGGCTCTGTCTAAGCAGC
Tat-S68A	Reverse	CCGCTTGGTGTGTCTGAGAGTTCT
Tat-S70A	Forward	CCAAGCGTCCCTGGCTAAGCAGCCTACATCT
Tat-S70A	Reverse	CCAGGGACGCTTGGTGTGTCTGAG
Tat-T74A	Forward	TGTCTAAGCAGCCTGCTTCTCAGTCTCGCG
Tat-T74A	Reverse	CAGGCTGCTTAGACAGGGACGCT
Tat-S75A	Forward	CTAAGCAGCCTACAGCTCAGTCTCGCGGC
Tat-S75A	Reverse	CTGTAGGCTGCTTAGACAGGGACGCTT
Tat-S77A	Forward	AGCCTACATCTCAGGCTCGCGGCGACCC
Tat-S77A	Reverse	CCTGAGATGTAGGCTGCTTAGACAGGGAC
Tat-T82A	Forward	CGCGGCGACCCAGCTGGACCTAGGGAGG
Tat-T82A	Reverse	CTGGGTCGCCGCGAGACTGAGAT
shFBXO45	Forward	CCGGACTTCCAAAGGTCTGCTTATACTCGAGTATAAGCAGACCTTTGGAAGTTTTTTG
shFBXO45	Reverse	AATTCAAAAAACTTCCAAAGGTCTGCTTATACTCGAGTATAAGCAGACCTTTGGAAGT

### Co-immunoprecipitation

After transfection with the indicated plasmids, HEK293T cells were harvested at 48 h and washed twice with cold PBS. Subsequently, the cells were dissociated in lysis buffer at 4°C for 30 min. The lysates were then pre-cleared by centrifugation at 10,000 × *g* for 30 min at 4°C and incubated with agarose beads conjugated to the specified antibody at 4°C overnight. Following this step, the mixtures underwent six washes using cold washing buffer (20 mM Tris, pH 7.5, 100 mM NaCl, 0.1 mM EDTA, and 0.05% Tween-20). Finally, the bound proteins were eluted in elution buffer (0.1 M glycine-HCl, pH2.0) and detected through immunoblotting using specific antibodies.

### Immunobloting analysis

The HEK293T cells were harvested 48 h post-transfection and lysed in 1× loading buffer (0.08M Tris, pH 6.8, 2.0% SDS, 10% glycerol, 0.1M dithiothreitol, and 0.2% bromophenol blue). Subsequently, the proteins were separated by a 12% SDS-PAGE gel and transferred onto polyvinylidene fluoride membranes. Then, the membranes were incubated with primary antibodies specific to the indicated proteins, followed by secondary antibodies. Afterward, the membranes underwent three washes with Tris-buffered saline–Tween (TBST) before being incubated with chemiluminescent HRP substrate (Millipore, Burlington, MA, USA) as per the manufacturer’s instructions. Finally, the bands were visualized using a multi-functional number imaging system (Azure CYCLOUD).

The antibodies used in this study are as follows: Rabbit polyclonal anti-HA(SG77) (#715500, Thermo, Waltham, MA, USA), Mouse monoclonal anti-Myc (clone 4A6) (#05–724, Millipore), Mouse monoclonal anti-Flag (M2) (#F1804, Sigma, St. Louis, MO, USA), Mouse monoclonal anti-β-Actin (#A00702, GenScript Corporation, PISCATAWAY, NJ, USA), Mouse monoclonal anti-p24 (catalog no. 1513; AIDS Research and Reference Reagents Program [ARRRP], USA), Rabbit polyclonal anti-FXBO45 (#Bs-13150R, Bioss), Rabbit polyclonal anti-p62 (#18420–1-AP, Proteintech, Rosemont, IL, USA), Mouse monoclonal anti-Tat (#02–001, Santa Cruz Biotechnology, Heidelberg, Germany), Goat anti-Mouse IgG (H + L) Cross-Adsorbed Secondary Antibody, Alexa Fluor 488 (#A11001, Thermo), Goat anti-Rabbit IgG (H + L) Cross-Adsorbed Secondary Antibody, Alexa Fluor 568 (#A11011, Thermo), Peroxidase AffiniPure Goat Anti-Rabbit IgG (H  +  L) (#AP132P, Millipore), and Peroxidase AffiniPure Goat Anti-Mouse IgG (H  +  L) (#AP124P, Millipore).

### Inhibitor treatment

To explore the mechanism underlying FBXO45-mediated degradation of Tat, we performed an inhibition assay using either MG132 (C2211, Sigma) or BafA1(HY-100558, MedChemExpress, Shanghai, China), or MLN7243 (S8341, Selleckchem, Houston, TX, USA). At 32 h post-transfection with indicated plasmids, HEK293T cells were treated with a 10 µM concentration of the proteasome inhibitor MG132, 10 nM concentration of the autophagy inhibitor BafA1, or 0.5 µM concentration of the ubiquitin-activating enzyme inhibitor MLN7243, whereas dimethyl sulfoxide was used as a negative control for another 16 h. Subsequently, the cells were harvested and subjected to immunoblotting analysis.

### CHX chase assay

To evaluate the steady-state levels of Tat in the presence or absence of FBXO45, a CHX chase assay was conducted. HEK293T cells were transfected with the indicated plasmids. Twenty-four hours post-transfection, the cells were treated with CHX (S7418, Selleckchem) at a final concentration of 100 µg/mL. The cells were harvested at the indicated time points and analyzed by immunoblotting assays.

### Confocal microscopy

For localization of Tat and FBXO45, Hela cells were transfected with Tat-Myc and FBXO45-HA constructs. Prior to fixation, the cells were treated with BafA1 for 16 h to prevent degradation, followed by fixation in 4% paraformaldehyde at room temperature for 15 min. After washing with PBS, permeabilization was performed using 0.1% Triton X-100 for 5 min, followed by another wash in PBS. Subsequently, blocking was performed using 2% BSA for 1 h before incubating the cells at room temperature for an additional 2 h with mouse anti-Myc and rabbit anti-HA antibodies (both diluted at a ratio of 1:1,000). Following a wash step, Goat anti-Mouse IgG was applied to the cells and incubated at room temperature for another hour. Finally, after washing with cold PBS, cell analysis was conducted using a laser scanning confocal microscope (LZM710, Carl Zeiss, Oberkochen Germany).

### RNA isolation and real-time RT-qPCR

The RNA was extracted using the Trizol Reagent (15596–026, Thermo) following the manufacturer’s protocol. Subsequently, DNase treatment with RQ1 RNase-free DNase (M6101, Promega) was performed to eliminate DNA contamination. For cDNA synthesis, the Transcriptor First Strand cDNA Synthesis kit (MR05101M, Monad, Wuhan, China) was employed. A total amount of 250–1,000 ng of total RNA served as a template for each cDNA synthesis reaction. RT-qPCR analysis was conducted on the Roche 480 instrument using the MonAmp ChemoHS qPCR Mix (MQ00401S, Monad). The relative levels of indicated genes were calculated using the 2^ΔΔCt method, normalized to GAPDH. The primer sequences used for RT-qPCR are listed in Table 2.

**TABLE 2:** Primers used for RT-qPCR in this study

Primer name	Primer direction	Sequence (5’−3’)
Gag-RT-F	Forward	CTGAAGCGCGCACGGCAA
Gag-RT-R	Reverse	GACGCTCTCGCACCCATCTC
Tat-RT-F	Forward	AGCCTTGGAAGCACCCTG
Tat-RT-R	Reverse	GCCTTTGTCATGAAGCACAC
PAM-RT-F	Forward	GGATGATACCACCAGGAACTCAG
PAM-RT-R	Reverse	GCCTATCTGCTCACTCTGAAGG
CUL1-RT-F	Forward	GCCTGGAATTATATAAACGAC
CUL1-RT-R	Reverse	CATATATTCCTTTTCGTCCTTC

### Cells and transfection

The adherent cell lines, the human embryonic kidney 293T (HEK293T) (catalog no. CRL-11268), HeLa (catalog no. CCL-2), and TZM-bl (catalog no. PTA-5659) were procured from the American Type Culture Collection (ATCC; Manassas, VA, USA), and cultured in Dulbecco’s modified Eagle’s medium (DMEM, 11995065, Thermo) with 10% fetal bovine serum (FBS; ST30-3302, PAN Seratech, Aidenbach, Germany). The HIV-1 latent cell line C11 was a kind gift from H. Z. Zhu (The College of Life Science, Fudan University). The MT-4 (catalog no. 120) cells were obtained from the AIDS Research and Reference Reagents Program, Division of AIDS, NIAID, NIH. Jurkat cells (ATCC catalog no. TIB-152), C11 cells, and MT-4 cells were cultured in RPMI 1640 medium supplemented with 10% FBS and Penicillin-streptomycin Solution (03–031-1B, Biological Industries, Israel). All cell lines were maintained at a temperature of 37°C in a humidified atmosphere containing 5% CO_2_.

The HEK293T cells seeded in 12-well plates at a monolayer with the density of 2.5 × 105 cells/well were transfected with indicated plasmids using Lipofectamine 2000 Reagent (#11668019, Thermo) following the manufacturer’s protocol. For siRNA transfection, Lipofectamine RNAiMAX Reagent (#13778150, Thermo) was employed.

### siRNA synthesis

The knockdown of endogenous FBXO45 and p62 was achieved using short interfering RNA (siRNA) specifically targeting FBXO45 and p62, as well as a non-specific control, which was procured from RiboBio (Guangzhou, China). The siRNA sequences are as follows: siFBXO45 (sense, 5′-CAGAUAGGAGAAAGAAUUCGAUU-3′, and antisense: 5′-UCGAAUUCUUUCUCCUAUCUGUU-3′); sip62 (sense, 5′-CGAGGAAUUGACAAUGGCCAUUU-3′, and antisense: 5′-AUGGCCAUUGUCAAUUCCUCGUU-3′); siCUL1 (sense, 5′-CUAGAUACAAGAUUAUACAUGCGUU-3′, and antisense: 5′-CGCAUGUAUAAUCUUGUAUCUAGUU-3′); and siPAM (sense, 5′-CCCGAGAUCUUGGGAAUAAUU-3′, and antisense: 5′-UUAUUCCCAAGAUCUCGGGUU-3′).

### Stable cell line construction

To generate a stable cell line for FBXO45 knockdown, the shRNA targeting FBXO45 was subcloned into the pLKO.1-puro shRNA vector. Replication-defective lentiviruses pseudotyped with vesicular stomatitis virus G protein were generated using a four-plasmid co-transfection method. Subsequently, C11 cells were infected with either the control lentivirus or the lentivirus harboring FBXO45-targeting shRNA. To construct MT-4 cells stably expressing FBXO45, a full-length FBXO45 sequence was cloned into the lentiviral expression vector pLVX-IRES-neo (Clontech Laboratories Inc., San Francisco, CA), and lentivirus overexpressing FBXO45 produced by HEK293T cells were used to infect MT-4 cells. After 48 h of infection, puromycin (1 µg/mL) was added to the culture medium for selection purposes. Five to 7 days later, immunoblotting analysis was performed to assess the expression of FBXO45.

### Luciferase assay

TZM-bl cells, derived from HeLa cells and containing an integrated HIV-1 LTR promoter, were infected with HIV-1, or HEK293T cells were transfected with HIV-1 LTR-luciferase construct plus indicated plasmids for 48 h. Subsequently, a dual-luciferase reporter assay was performed using the Promega Dual-Luciferase Reporter Assay System (Promega) according to the manufacturer’s instructions and measured using the GloMax 20/20 Luminometer (Promega).

### Flow cytometry

Flow cytometry (FACSCalibur; BD, Franklin Lakes, NJ, USA) was employed to assess the antiviral effect of MT-4 cells stably overexpressing FBXO45 upon infection with VSV-G pseudotyped HIV-1 NL4-3-GFP virus, as well as the latent C11 cells reactivation, by quantifying the percentage of GFP-positive cells.

### Data statistics

The data in this study represented three independent repetitions of each experiment and are shown as the mean ± standard deviation (SD). Statistical significance was assessed using Student’s *t*-test or ANOVA. *P < 0.05, **P < 0.01, ***P < 0.001, and ns denotes no significance.

## Data Availability

All data sets generated for this study are within the paper. The mass spectrometry proteomics data have been deposited in the ProteomeXchange Consortium (https://proteomecentral.proteomexchange.org) with the accession code PXD059409.
